# Endovascular Treatment of a Symptomatic Vertebral Artery Aneurysm in a Puerperal Patient with Neurofibromatosis Type 1—A Case Report and Review of the Literature

**DOI:** 10.3390/diseases13070226

**Published:** 2025-07-18

**Authors:** Nikola Mirkovic, Marko Prokic, Nikola Prodanovic, Tamara Nikolic Turnic, Nikola Andric, Tijana Prodanovic, Neda Arsenijevic, Ivan Simic, Dragan Knezevic, Aleksandar Matic

**Affiliations:** 1 Vascular Surgery Center, University Clinical Center Kragujevac, Zmaj Jovina 30, 34000 Kragujevac, Serbia; drnikolamirkovic@gmail.com (N.M.); dragankg984@gmail.com (D.K.); 2 Department of Surgery, Faculty of Medical Science, University of Kragujevac, Svetozara Markovica 69, 34000 Kragujevac, Serbia; nikolaprodanovickg@gmail.com (N.P.); andricnikola91kg@gmail.com (N.A.); maticaleksandar@gmail.com (A.M.); 3 General and Thoracic Surgery Clinic, University Clinical Center Kragujevac, 34000 Kragujevac, Serbia; prokmarko@yahoo.com; 4 Clinic for Orthopedic and Trauma Surgery, University Clinical Center Kragujevac, Zmaj Jovina 30, 34000 Kragujevac, Serbia; 5 Department of Pharmacy, Faculty of Medical Sciences, University of Kragujevac, Svetozara Markovica 69, 34000 Kragujevac, Serbia; 6 N.A. Semashko Public Health and Healthcare Department, F.F. Erismann Institute of Public Health, I.M. Sechenov First Moscow State Medical University, 119435 Moscow, Russia; 7 Department of Pediatrics, Faculty of Medical Sciences University of Kragujevac, University of Kragujevac, Svetozara Markovica 69, 34000 Kragujevac, Serbia; tijanaprodanovic86@gmail.com; 8 Center for Neonatology, Pediatric Clinic, University Clinical Center Kragujevac, Zmaj Jovina 30, 34000 Kragujevac, Serbia; 9 Department of Gynecology and Obstetrics, Faculty of Medical Sciences, University of Kragujevac, Svetozara Markovica 69, 34000 Kragujevac, Serbia; velickovicneda@gmail.com; 10 Clinic of Gynecology and Obstetrics, University Clinical Center Kragujevac, Zmaj Jovina 30, 34000 Kragujevac, Serbia; 11 Department of Internal Medicine, Faculty of Medical Sciences, University of Kragujevac, Svetozara Markovica 69, 34000 Kragujevac, Serbia; ivansimickg@gmail.com; 12 Clinic for Cardiology, University Clinical Center Kragujevac, Zmaj Jovina 30, 34000 Kragujevac, Serbia

**Keywords:** vertebral artery aneurysm, neurofibromatosis type I, puerperium, flow diverter, stent graft

## Abstract

Introduction: Primary extracranial vertebral artery aneurysms are sporadic in the general population. They are uncommon in individuals with neurofibromatosis type 1. During pregnancy or in the puerperium, the risk of aneurysm rupture in individuals with neurofibromatosis type 1 and extracranial aneurysms is elevated. Rupture of a vertebral artery aneurysm is an emergency condition and can be fatal. Case presentation: We present the case of a 33-year-old woman in the puerperium with neurofibromatosis type 1 who had a vertebral symptomatic artery aneurysm. During a previous hospitalization, two months before the treatment of the vertebral aneurysm, a same-sided aneurysm of the thyrocervical trunk was successfully treated with endovascular coiling because of aneurysm rupture. In this case report, the vertebral artery aneurysm was successfully managed using a flow diverter stent graft. Conclusions: This is the first reported case of a successfully treated symptomatic vertebral artery aneurysm with a flow diverter stent graft in a patient with neurofibromatosis type 1 during the early puerperium. Endovascular treatment with a stent graft is a minimally invasive, safe, and effective treatment for patients with vertebral artery aneurysms. Early diagnosis of non-ruptured vertebral artery aneurysms is a crucial as well as appropriate treatment, which should be undertaken in a timely manner to prevent serious complications or a fatal outcome.

## 1. Introduction

Aneurysms of the extracranial segment of the vertebral artery are exceedingly rare in the general population [1]. Approximately 25% of cerebrovascular strokes originate from the vertebrobasilar system [1]. These strokes are most frequently caused by vertebral artery pathologies, including aneurysms, dissections, atherosclerosis, fibromuscular dysplasia, neurofibromatosis, arteritis, and trauma [1,2]. Vertebral artery aneurysms (VAAs) can lead to significant complications within the vertebrobasilar system, resulting in severe and potentially life-threatening consequences. VAAs may be complicated by aneurysm rupture, brain ischemia due to embolization, and compression of surrounding structures [1]. The rupture of a VAA can result in hemorrhagic shock, hemothorax, and other life-threatening conditions. These complications substantially increase morbidity and mortality rates among patients with VAAs [1].

Endovascular options for treating aneurysms or vessel injuries include coiling, trapping, and covered stents, each with distinct risks and benefits. Coiling, the most common, involves deploying coils to fill the aneurysm sac, potentially leading to incomplete occlusion or regrowth. Trapping, or parent artery occlusion, involves blocking the affected vessel, making it effective for hemorrhage control but risky if collateral circulation is inadequate. Covered stents offer a less invasive alternative, sealing the leak while preserving the parent artery, but may have long-term risks of recurrence [2,3,4,5,6]. Choosing the right endovascular treatment option requires a careful evaluation of these factors and a thorough discussion with the patient’s healthcare team.

Neurofibromatosis type 1 (NF-1) is a genetic disorder caused by a mutation in the NF-1 gene on chromosome 17. It is a hereditary condition that is relatively rarely diagnosed in clinical practice. The NF-1 gene encodes the production of a specific protein called neurofibromin. The significance of neurofibromin lies in its role in regulating cell growth by inhibiting the Ras oncogene. If a mutation occurs in the NF-1 gene, it can lead to the formation of tumours called neurofibromas [3,4]. Patients with NF-1 typically exhibit characteristic clinical features, including neurofibromas in multiple organs, cutaneous neurofibromas, macrocephaly, syringomyelia, kyphoscoliosis, and cutaneous signs such as Lisch nodules, café-au-lait spots, optic gliomas, pigmented iris, and hamartomas [4,5,6]. It is important to note that individuals with NF-1 may have abnormal blood vessels. As a result of the impaired function of the NF-1 gene, vascular endothelial cells may develop aneurysms, stenoses, occlusions, aortic coarctation, and arteriovenous malformations due to weakened blood vessel walls, potentially leading to significant complications [2,3,7,8].

During pregnancy, difficulties arise because of hormonal changes in the levels of estrogen and progesterone in the plasma, leading to alterations in the structure of blood vessels. Elevated levels of these hormones cause changes in the media and intima layers, resulting in the weakening of the blood vessel walls [9]. These hemodynamic changes are most pronounced during the third trimester of pregnancy when maternal blood volume, heart rate, blood pressure, stroke volume, and cardiac output increase [9,10]. Arterial wall tension rises, along with increased shear stress [10]. Significant hemodynamic changes particularly occur during labour and the early postpartum period. With each uterine contraction, approximately 500 mL of venous blood is pushed back [11]. During pushing and delivery of the fetus, there is intense pain and marked fluctuations in central venous pressure. In the third stage of labour, the delivery of the placenta results in an “autotransfusion” of an additional 500 mL of blood from the uteroplacental circulation returning to the maternal circulation [11]. Increased central venous pressure and cardiac output characterize these hemodynamic changes and pose an elevated risk of aneurysm rupture [11]. Therefore, strict blood pressure and heart rate control during labour and the early puerperium are crucial for reducing the risk of rupture [11]. Pregnancy is not associated with an increased incidence of aneurysm formation, but with an elevated risk of rupture [12]. They also increased the risk of aneurysm rupture during the early puerperium [13]. Hence, hemodynamic stability is paramount in pre-venting aneurysm rupture during pregnancy and the puerperium in affected individuals. Regular monitoring of blood pressure and pulse, along with necessary adjustments, is essential. Given the heightened risk of aneurysm rupture, it is necessary to manage pregnant women with aneurysms through a multidisciplinary approach in a tertiary care centre, involving obstetricians, vascular surgeons, cardiologists, nephrologist, and anesthesiologists. Regular monitoring of the pregnant woman, including routine cardiac and aneurysm ultrasounds, is essential [11].

If such specific patients with VAAs are not recognized, adequately diagnosed, and treated with appropriate surgical or endovascular interventions, the consequences can be catastrophic and life-threatening. It is crucial promptly identify ruptured extracranial artery aneurysms if they are suspected and start urgent treatment for these patients. An early diagnosis of non-ruptured aneurysms is essential to facilitate timely intervention and prevent serious complications that, in extreme cases, may cause fatal outcomes.

## 2. Case Presentation

In this article, we present the case of a 33-year-old woman with a primary extracranial symptomatic aneurysm of the left VAA without rupture, with neck pain. This report highlights a rare but potentially life-threatening complication. The patient was admitted to the Centre for Vascular Surgery, specifically to the Department of Endovascular Surgery. Two months earlier, during a previous hospitalization, she suffered an ipsilateral thyrocervical trunk ruptured aneurysm on the same side, accompanied by left-sided haemothorax and haemorrhagic shock three days after a Cesarean section. This condition was successfully treated with an emergency endovascular coiling intervention. After the patient’s recovery from the ruptured thyrocervical aneurysm (TCA) and adequate preoperative preparation, we proceeded with endovascular treatment of the ipsilateral VAA, which had been previously confirmed angiographically.

The patient had a medical history of NF-1, which manifested with dermal and spinal neurofibromas, café-au-lait spots, and kyphoscoliosis of the cervical and thoracic spine. There was no trauma to the left supraclavicular region, clavicle fracture, or central venous catheter placement. The patient did not use tobacco. The basic coagulation profile was within normal limits. Other comorbidities included arterial hypertension. The patient was on daily therapy with beta-blockers, angiotensin-converting enzyme inhibitors, and antiplatelet therapy. There was a positive family history of Neurofibromatosis type 1, as the patient’s mother was also affected. Clinical examination revealed no neurological abnormalities. Peripheral pulses were easily palpable at anatomical reference sites.

**Figure 1:**
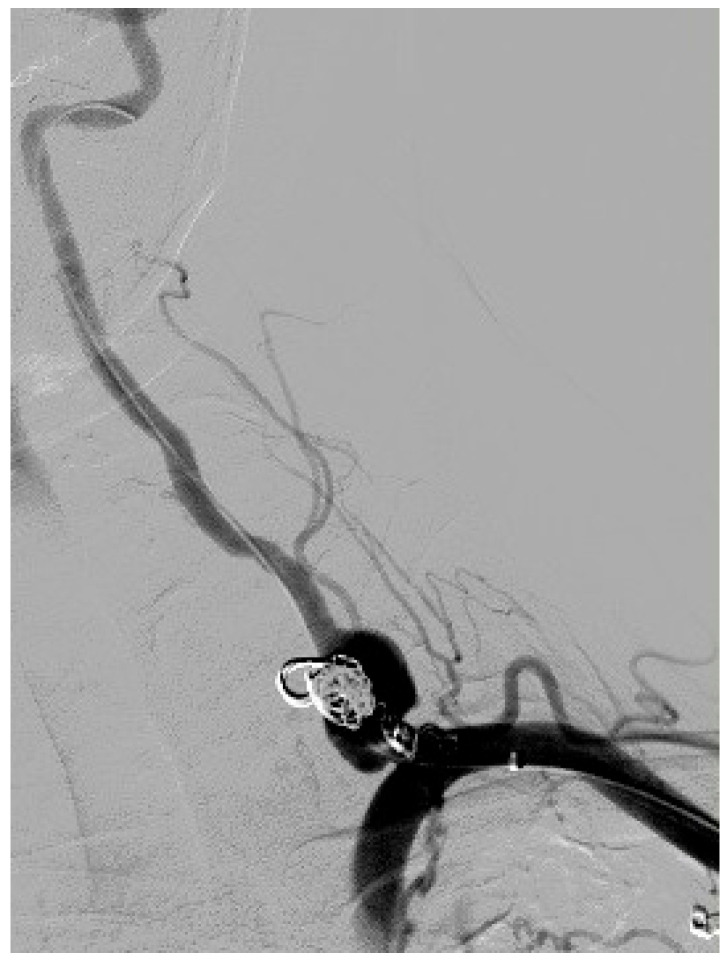
Digital subtraction angiography of the left vertebral artery aneurysm without extravasation of contrast. The ipsilateral thyrocervical trunk ruptured aneurysm had been previously occluded by coiling embolization and completely excluded from circulation.

The precise anatomical location of the VAA had been identified through prior digital subtraction angiography (DSA). A follow-up DSA confirmed the left VAA without contrasting extravasation. The ipsilateral thyrocervical trunk aneurysm had been completely excluded from circulation and occluded by coiling embolization during the previous hospitalization because of the rupture of the TCA (Figure 1). The endovascular procedure for the VAA was indicated and planned.

The patient and family were informed about the risks associated with the intervention and the potential consequences of leaving the VAA untreated. Following complete preoperative preparation, the procedure was performed under local anesthesia using a transfemoral approach through the right femoral artery to access the left VAA.

**Figure 2:**
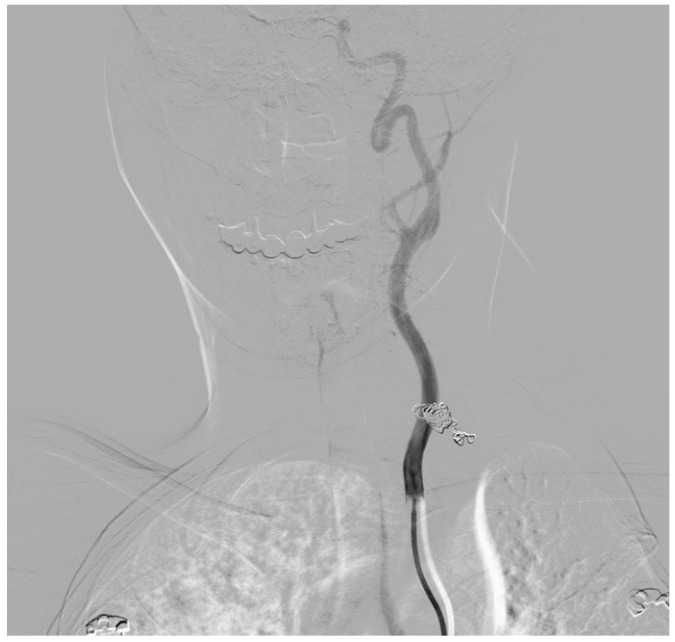
Post-endovascular digital subtraction angiography showing successful complete exclusion of the left vertebral artery aneurysm from circulation while preserving flow through the vertebral artery.

After catheter positioning, a flow diverter stent graft (Derivo Device Acandis, GmbH, Pforzheim, Germany) was successfully placed in the left VAA. Post-stenting angiography confirmed complete exclusion of the left VAA from circulation while preserving flow through the left VAA (Figure 2). Finally, follow-up DSA confirmed the success of the procedure. The femoral access site was closed using a mechanical closure device (Angio-Seal 6 VIP, Terumo^®^, Tokyo, Japan).

During the procedure, the patient experienced no complications. The patient was monitored for the first 24 postoperative hours with no significant postoperative bleeding. The femoral artery puncture site was without hematoma and well healed. Postoperative monitoring continued regularly. Control ultrasonography confirmed stent graft patency in the vertebral artery, with the VAA successfully excluded from circulation and no evidence of an endo leak.

All peripheral pulses remained palpable and normal following the intervention. Cranial nerve function remained intact after the procedure, and the patient exhibited no hemodynamic or respiratory disturbances. The recovery was smooth and uneventful. The patient was discharged from the hospital on dual antiplatelet therapy for three months (ticagrelor 90 mg twice daily and acetylsalicylic acid 100 mg/day).

This case report highlights a very rare but potentially life-threatening complication of VAA. Endovascular treatment of VAA using a flow diverter stent graft proved to be a minimally invasive, safe, and effective method. The patient was discharged 2 days after the intervention (Table 1).

## 3. Discussion

Primary extracranial VAAs typically occur in patients with hereditary connective tissue disorders [1,2,3,4,5]. Although VAAs are extremely rare in the general population, they carry a high risk of serious complications. The most severe complication is an aneurysm rupture, which presents with sudden, intense neck pain, headaches, and neck hematoma, potentially causing compression of surrounding structures. Hematoma formation can lead to nerve damage and airway compression, while bleeding into the thoracic cavity may cause haemothorax—a rare but severe complication that impairs respiratory function—and further deteriorates the patient’s condition [10,11,12,13,14]. Uncontrolled bleeding and haemorrhagic shock significantly increase morbidity and mortality in these patients [2,6,15]. In patients with VAAs, cerebrovascular events may occur because of aneurysm thrombosis or embolization from the aneurysm sac to the brain. Patients with extracranial aneurysms are usually asymptomatic. These aneurysms, often small and in the carotid, vertebral, subclavian arteries, or thyrocervical trunk, are frequently discovered incidentally. Small aneurysms are usually observed [7,8,9,10,16,17]. However, larger aneurysms may become symptomatic, typically manifesting during puberty or early adulthood. Patients with NF-1 who present with neck pain, radiculopathy, cervical hematoma, or hemithorax should be suspected of having VAA, TTA, or intercostal artery aneurysm [11,12,18].

The association between neurofibromatosis and extracranial aneurysms is often under-recognized but well-documented. Raising awareness of this rare yet high-risk condition is crucial, as early diagnosis can prevent severe morbidity and mortality [19]. For example, in pregnant and postpartum women, elevated levels of oestrogen and progesterone cause arterial wall remodelling, leading to fibro-muscular dysplasia in the tunica media and elastic fibre fragmentation [20]. These vascular changes increase the risk of aneurysm rupture. Multiparity may further elevate this risk of aneurysm rupture. Pregnant and postpartum women are at an increased risk of aneurysm rupture compared to their non-pregnant counterparts or those not in the puerperium [21]. Therefore, preventive screening and cardiovascular evaluation are essential for high-risk patients with hereditary connective tissue disorders and aneurysmal disease. This is relevant during pregnancy and the puerperium in patients with NF-1 patients, where the likelihood of cardiovascular events could be increased.

Previous reports in the literature have documented the successful treatment of extracranial aneurisms in patients with NF-1 using endovascular intervention [3,22,23]. Endovascular treatment of VAAs can be performed either electively or emergently. Compared to traditional surgical resection, endovascular treatment significantly reduces the risk of complications, such as nerve injury (e.g., Horner’s syndrome and vocal cord paralysis), vascular and lymphatic damage, and risks associated with general anesthesia [24].

We present the first reported case of a successfully treated VAA using a flow diverter stent graft in a patient with NF-1 during the early puerperium. We opted for a flow diverter stent graft because of its simplicity and effectiveness in excluding the aneurysm from circulation while preserving ipsilateral flow to the basilar artery. Given the patient’s previous ipsilateral rupture of a thyrocervical aneurysm, which was treated with endovascular coiling, and a large residual hematoma on the left side of the neck that had not fully resolved, an endovascular approach was preferred. Traditional surgical intervention posed a high risk because of challenging access through altered tissue resulting from the previous rupture and hematoma. Endovascular treatment provided a safer and more straightforward option. The procedure was smooth and uneventful, under local anaesthesia, and the patient experienced no post-intervention complications. This patient case demonstrates that a flow diverter stent graft for VAA, when workable, is a safer, less invasive, and more effective treatment option compared to open conventional surgery.

The literature suggests that endovascular procedures are associated with fewer complications, lower morbidity, and reduced mortality compared to traditional surgical treatment [24]. Endovascular treatment has proven to be an excellent and effective option for managing complex vertebral artery pathology, with favourable outcomes and lower complication rate than conventional surgery. However, complications can still occur during endovascular interventions because of wire, catheter, or stent manipulation within the aneurysm [15]. Potential risks include cerebral embolization, arterial dissection, or an endo leak—particularly in tortuous vertebral arteries with stent malposition [1]. To prevent complications, dual antiplatelet therapy is essential after deploying a flow diverter stent in a primary vertebral artery aneurysm. Conventional surgical resection remains the gold standard when endovascular treatment is not workable or technically unsuccessful because of aneurysm morphology or procedural limitations. However, with advancements in technology, endovascular procedures have significantly improved success rates, making minimally invasive techniques comparable to open surgery. The application of new techniques and technologies has made the diagnosis and treatment of vascular diseases easier and more effective.

There is ongoing debate regarding the optimal treatment strategy for extracranial aneurysms, as both surgical and endovascular procedures are technically challenging and complex, yet effective and successful. Endovascular treatment has emerged as a preferred approach for vertebral artery aneurysms, with lower mortality and morbidity rates compared to conventional open surgical intervention [24]. This procedure is safe, minimally invasive, beneficial, and effective, with fewer complications, shorter operative times, and improved quality of life compared to open conventional surgery [20]. Endovascular treatment is helpful for aneurysms in anatomically challenging locations.

There are no previous reports about the simultaneous presence of VAA and TTA in the literature. Given that, we present a case of ipsilateral VAA and TTA successfully treated with a 2-stage endovascular intervention in a patient with NF-1 during the early puerperium. The successful management of aneurysms underscores the effectiveness of endovascular techniques in treating complex vascular conditions, particularly in high-risk patients in extremely rare conditions.

## 4. Conclusions

VAAs in patients with NF-1 are rare but potentially life-threatening. This is the first reported case of a successfully treated symptomatic vertebral artery aneurysm using a flow diverter stent graft in an NF-1 patient during the early puerperium. Endovascular stent grafting is a minimally invasive, safe, and effective approach for vertebral artery aneurysms, particularly in patients with complex comorbidities. Early diagnosis of non-ruptured aneurysms is crucial, and timely intervention is necessary to prevent serious complications, including aneurysm rupture and patient mortality.

## Figures and Tables

**Table 1:** Patient’s clinical course.

Timeline	Patient’s Clinical Course	Intervention
0 day	Admission to Gynecology and Obstetrics Clinic	Cesarean section (CS)
3 days after CS	Admission to Centre for Vascular Surgery	Left sided ipsilateral thyrocervical trunk ruptured aneurysm
3 days after CS	Centre for Vascular Surgery	emergency endovascular coiling intervention
2 months after CS	Admission to Centre for Vascular Surgery	Left sided endovascular treatment (ET) of the ipsilateral VAA
2 days after ET	Discharge from Centre for Vascular Surgery	Monitoring and dual antiplatelet therapy

CS—Cesarean section; ET—endovascular treatment.

## Data Availability

All results are presented in this manuscript.
